# Neonatal *Ureaplasma parvum* meningitis complicated with subdural hematoma: a case report and literature review

**DOI:** 10.1186/s12879-021-05968-1

**Published:** 2021-03-17

**Authors:** Canyang Zhan, Lihua Chen, Lingling Hu

**Affiliations:** Departments of Neonatology, The Children’s Hospital, Zhejiang University School of Medicine, National Clinical Research Center for Child Health, Hangzhou, China

**Keywords:** Neonate, *Ureaplasma parvum*, Meningitis, Subdural hematoma, Metagenomic next-generation sequencing (mNGS)

## Abstract

**Background:**

Neonatal meningitis is a severe infectious disease of the central nervous system with high morbidity and mortality. *Ureaplasma parvum* is extremely rare in neonatal central nervous system infection.

**Case presentation:**

We herein report a case of *U. parvum* meningitis in a full-term neonate who presented with fever and seizure complicated with subdural hematoma. After hematoma evacuation, the seizure disappeared, though the fever remained. Cerebrospinal fluid (CSF) analysis showed inflammation with CSF pleocytosis (1135–1319 leukocytes/μl, mainly lymphocytes), elevated CSF protein levels (1.36–2.259 g/l) and decreased CSF glucose (0.45–1.21 mmol/l). However, no bacterial or viral pathogens in either CSF or blood were detected by routine culture or serology. Additionally, PCR for enteroviruses and herpes simplex virus was negative. Furthermore, the CSF findings did not improve with empirical antibiotics, and the baby experienced repeated fever. Thus, we performed metagenomic next-generation sequencing (mNGS) to identify the etiology of the infection. *U. parvum* was identified by mNGS in CSF samples and confirmed by culture incubation on mycoplasma identification medium. The patient’s condition improved after treatment with erythromycin for approximately 5 weeks.

**Conclusions:**

Considering the difficulty of etiological diagnosis in neonatal *U. parvum* meningitis, mNGS might offer a new strategy for diagnosing neurological infections.

## Background

Neonatal meningitis is a severe infection of the central nervous system (CNS) with high morbidity and mortality and can lead to serious neurological complications or long-term disabilities [1, 2]. *Ureaplasma spp.*, including *Ureaplasma parvum* (*U. parvum)* and *Ureaplasma urealyticum* (*U. urealyticum)*, are rare pathogens in neonatal CNS infection. It has been indicated that *Ureaplasma spp.* are associated with adverse neonatal outcomes, such as congenital pneumonia, bronchopulmonary dysplasia and perinatal death [3, 4]. However, there are few reports about *U. parvum* as an invasive organism in neonatal meningitis. Herein, we describe a full-term infant who developed *U. parvum* meningitis complicated with subdural hematoma.

## Case presentation

The male baby in this case was born by spontaneous vaginal delivery (gravida 2 para 2) at 40 weeks of gestation with a birth weight 3800 g. The pregnancy was uneventful. Half an hour after birth, the baby was admitted to the neonatal department with moaning and tachypnea. Ampicillin sulbactam was initiated due to the possibility of pneumonia. Laboratory tests showed leukocytosis (17.93–35.3 × 10^9^/l) and C-reactive protein (CRP: 0.1–11.2 mg/l). Although his symptoms improved, the baby had a fever on day 5 of hospitalization. The antibiotic was changed to cefotaxime, though the baby still had a mild fever on day 9 of hospitalization. Moreover, blood analysis revealed greatly elevated CRP (47.5 mg/l). MRI revealed right parietal temporal and bilateral occipital subdural hemorrhage, and the baby (9 days old) was transferred to our hospital due to subdural hemorrhage.

**Fig. 1:**
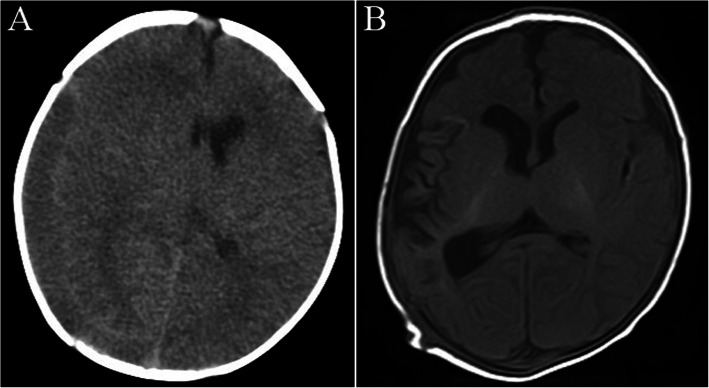
The change in imaging of the head during hospitalization. CT scan displayed a right parietal temporal hematoma with a midline shift (**a**). After subdural hematoma removal and external drainage of subdural hemorrhage, MRI revealed atrophy of the right cerebral hemisphere with cortical necrosis and mild dilatation of the third ventricle. No detection of intraventricular hemorrhage was found (**b**)

**Fig. 2:**
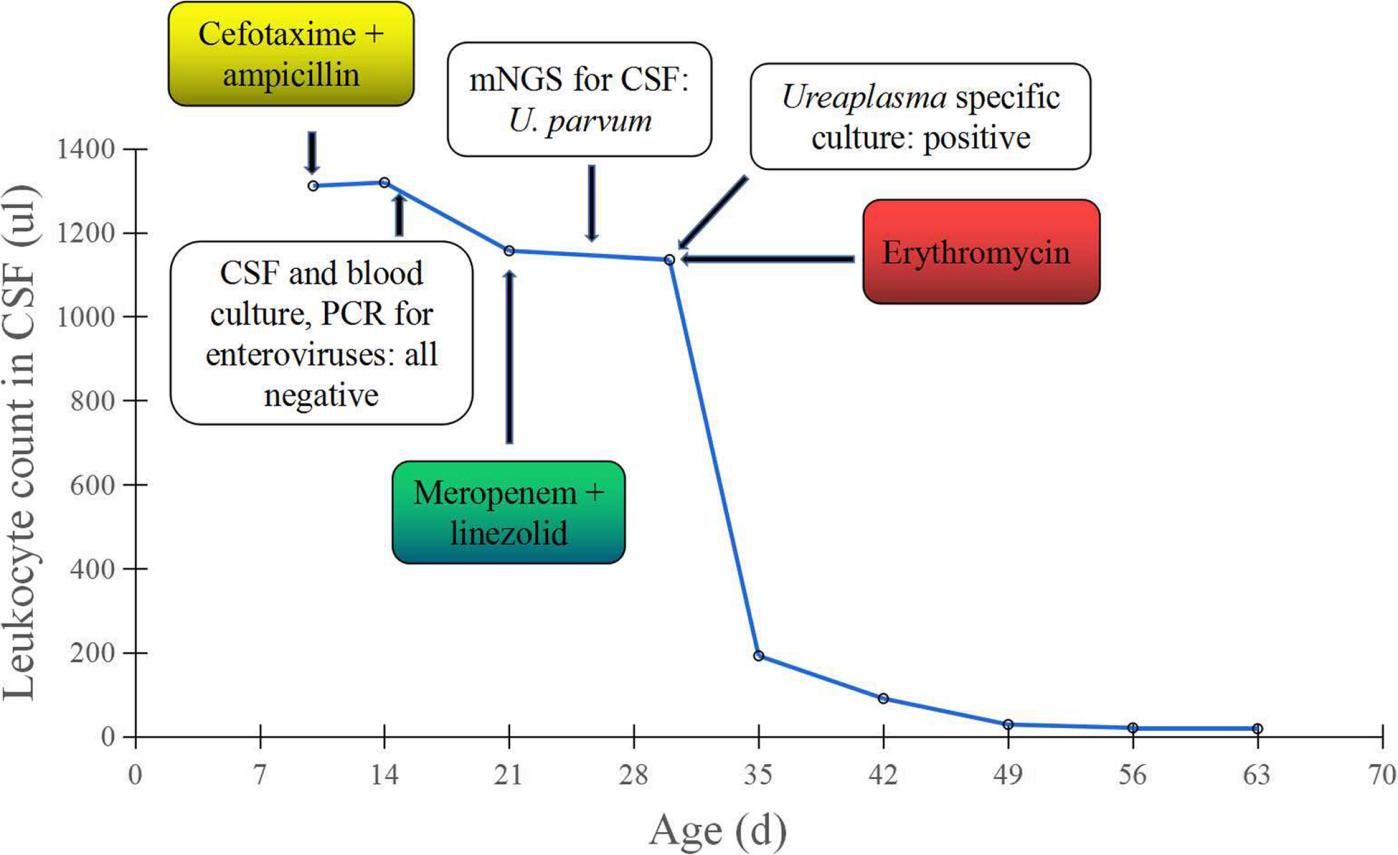
Change in leukocyte counts in CSF, and timeline of antibiotics and microbiology tests

On admission to our hospital, the baby still had a fever (37.7 °C) with tight fontanel. The disease progressed, and the baby developed seizures. The cerebrospinal fluid (CSF) cell count was 1311/l (88% lymphocytes); protein and glucose levels were 1.653 g/l and 1.21 mmol/l, respectively. CT scan displayed right parietal temporal hematoma with a midline shift (Fig. 1a). Intravenous empiric treatment for meningitis with cefotaxime and ampicillin was started, as was intracranial pressure relief (glycerol fructose), hemostasis and anticonvulsion therapy. Two days later, subdural hematoma removal and external drainage of the subdural hemorrhage were performed. Four days after the surgery, the baby had a recurrent fever. CSF analysis repetitively revealed pronounced inflammation of bacterial meningitis, as reflected by CSF pleocytosis (1135–1319 leukocytes/μl, 60–66% lymphocytes), CSF protein levels of 1.36–2.259 g/l and CSF glucose levels of 0.45–1.21 mmol/l. However, microbial diagnostics, including repetitive culture of the CSF and blood, were not informative. Additionally, PCR for enteroviruses and herpes simplex virus was negative. Because of the recurrence of symptoms, treatment with meropenem and linezolid was started based on the suspicion of resistant bacteria on day 12 of hospitalization. Furthermore, the CSF was collected and sent for pathogen detection by metagenomic next-generation sequencing (mNGS) (BGI, Shenzhen, China). *U. parvum* was detected, and CSF culture on mycoplasma identification medium (Jiangmen Kailin Trading Co., Ltd., China.) for *Ureaplasma spp.* was positive. The diagnosis was confirmed as *U. parvum* meningitis. Meropenem and linezolid were discontinued, and intravenous erythromycin (30 mg/kg/d bid) was started. His clinical symptoms improved quickly, and the CSF gradually normalized (Fig. 2). After 36 days, CSF culture was negative for *Ureaplasma spp.*, and mNGS revealed no *U. parvum* in the CSF; thus, erythromycin was stopped. During hospitalization, MRI revealed atrophy of the right cerebral hemisphere with cortical necrosis (Fig. 1b). Ultrasound before discharge showed a slight enlargement of lateral ventricles (body of left lateral ventricle: 0.64 cm; body of right lateral ventricle: 0.60 cm). The infant developed well without abnormal neurological signs at the follow-up when he was 5 months old.

## Discussion and conclusions

*Ureaplasma* belongs to the family Mycoplasmataceae, which is a common opportunistic pathogens of the urogenital tract with a colonization rate of 40–80%. The vertical transmission rate of *Ureaplasma* may range from 18 to 88% [5]. Since 2002, human *Ureaplasma spp.* have been divided into two groups: *U. parvum* (serovars 1, 3, 6, and 14) and *U. urealyticum*. (serovars 2, 4, 5, and 7 to 13) [6]. It is reported that both the prevalence and transmission of *U. parvum* in pregnant women are higher than those of *U. urealyticum* [7, 8]. *Ureaplasma spp.* has been associated with adverse neonatal outcomes, but little is known about CNS infection by these bacteria in neonates. Nonetheless, a growing number of studies have focused on the relationship between *Ureaplasma spp.* and CNS infection/inflammation [9, 10]. Some in vitro studies found evidence of *Ureaplasma*-driven apoptosis in human brain microvascular endothelial cells (HBMECs), which might ultimately result in blood-brain barrier breakdown and CNS inflammation [10].

*U. parvum* meningitis has rarely been reported in neonates. To the best of our knowledge, there are only five case reports of *U. parvum* neonatal meningitis in the English literature (Table 1) [11–15]. The presentation of *U. parvum* CNS infection in neonates is atypical and includes fever, irritability, floppy, seizure, apnea, bradycardia and intraventricular hemorrhage (IVH). The main complications reported are dilatation of the lateral ventricles, IVH (grade I - IV), and subdural hygromas. In particular, ventricular dilation or hydrocephalus was observed in all 6 cases. Three patients underwent ventriculoperitoneal (VP) shunt surgery. This appears to be the first case report of neonatal *U. parvum* CNS infection complicated by subdural hematoma. In addition, CSF changes in *Ureaplasma* meningitis were characterized by pleocytosis and significantly elevated CSF protein levels and reduced glucose levels (Table 2). Neither the clinical manifestation nor the CSF characteristics of *U. parvum* meningitis differed from those of meningitis due to other bacteria. The diagnosis of *U. parvum* CNS infection is challenging.

**Table 1:** Basic characteristics of neonatal meningitis caused by *U. parvum*

Author, year	Sex/Age (d)	Birth weight (g)	Gestation (weeks)	Presentation/Complication
Clifford V et al., 2010 [11]	Female/18	1630	30	fever, cardiovascular instability apnea, IVH (III), hydrocephalus
Biran V et al., 2010 [12]	NA/10	3500	39	fever, rhinitis, conjunctivitis, seizure, dilated ventricles
Glaser K et al., 2014	Female/28	946	26 + 3	weak and floppy, generalized muscular hypotonia, lack of tendon reflexes; hydrocephalus,
Keus AMJMH et al., 2019 [13]	Male/6	NA	Full term^a^	irritability, fever, seizure, ventricular dilation, ventriculitis, subdural collections
Wang Q et al., 2020 [14]	Male/11	3390	40	fever, floppy, seizure, hydrocephalus
Our case	Male/5	3800	40	fever, seizure, subdural hemorrhage, enlargement of lateral ventricles

^a^The exact number of weeks was not given in the article

**Table 2:** Diagnosis, treatment and prognosis of neonatal meningitis caused by *U. parvum*

Author, year	Identification	CSF parameters (before treatment for *U. parvum* meningitis)			Treatment		Prognosis
		Leukocytes (ul)	Protein (g/l)	Glucose (mmol/l)			
Clifford V et al., 2010 [11]	PCR	76 (18 polymorphonuclear cells and 58 lymphocytes)	2.86	< 1.2	ERY (30 mg/kg/d) × 3 d → ERY + CIP (20 mg/kg/d) × 3 weeks	VP shunt	developed appropriately at the age of 6 months
Biran V et al., 2010 [12]	PCR and culture	1610 (86% polymorphonuclear)	5.2	0.1	CIP × 7 weeks + THI × 3 weeks	VP shunt	developed appropriately at the age of 8 and 14 months
Glaser K et al., 2014	PCR	50–86 (60% lymphocytes)	5.78–10.26	undetectable	CAM × 2d → CHL × 3 weeks	VP shunt	recovered from severe developmental delay (tendon reflexes were improved), being able to sit unaided, to walk with little assistance and to comprehend simple linguistic requests at 16 month adjusted age
Keus AMJMH et al., 2019 [13]	PCR	1387	NA	NA	ERY (30 mg/kg/d) + CIP (20–25 mg/kg/d) × 5 weeks → AZI (12 mg/kg/d) + CIP (25 mg/kg/d) × 1 weeks	NA	normal age-related development at the age of 30 months
Wang Q et al., 2020 [14]	mNGS and PCR	880 (48% of neutrophils)	2.6	< 1.11	ERY (10 mg/kg/dose q6 h) × 4 weeks	NA	developed well without abnormal neurological signs during the follow-up until 18 months old. Hydrocephalus was improved at the age of 6 months.
Our case	mNGS and culture	1135–1319 (60–88% lymphocytes)	1.36–2.259	0.45–1.21	ERY (30 mg/kg/d) × 5 weeks	removal of subdural hematoma	developed well without abnormal neurological signs during the follow-up at 5 months old

*PCR* Polymerase chain reaction, *mNGS* Metagenomic next-generation sequencing, *CSF* Cerebrospinal fluid, *ERY* Erythromycin, *CIP* Ciprofloxacin, *VP* Ventriculoperitoneal, *THI* Thiamphenicol, *CAM* Clarithromycin, *CHL* Chloramphenicol, *AZI* Azithromycin, *NA* Not available

Without a cell wall, *Ureaplasma spp*. cannot be stained by Gram stain, and microbial diagnosis of *Ureaplasma spp*. requires special culture. In some labs, there is no special culture media for detecting *Ureaplasma spp.* in the CSF. Therefore, the diagnosis of *Ureaplasma* meningitis in neonates might often be delayed. At approximately 90%, PCR is considered to be more sensitive than culture for the detection of *Ureaplasma spp*. [16, 17]. However, in this case, both routine culture and PCR (for enteroviruses and herpes simplex virus) of the CSF were negative. We confirmed the microbe by mNGS, which has been applied as a novel strategy for detecting pathogens causing infectious diseases, especially neurological infections [18].

CSF mNGS is an unbiased approach that can diagnose infections due to viruses (DNA and RNA viruses), parasites, fungi and bacteria in a single test. Moreover, it can identify pathogens that are rare, novel or overlooked. mNGS has a higher sensitivity than culture for pathogens, and mNGS data are increasingly available for diagnosing the pathogen in neurological infections [19]. However, nucleic acid contamination from specimens or the environment might lead to false positives, making it challenging to interpret the results. Furthermore, it is expensive, with costs of approximately US $2000 [18]. In China, the cost is approximately US $600–1000, which is also more expensive than culture and PCR. In our opinion, mNGS is helpful for diagnosing intracranial infection when routine testing of microbes is negative and the infection is not improved by routine treatment.

The treatment of *U. parvum* infection meningitis in neonates is limited. There is no consensus regarding the choice of antibiotic, dosage, or duration of treatment. It has been reported that macrolides, chloramphenicol, fluoroquinolones and thiamphenicol can be used for *U. parvum* meningitis as either monotherapy or combination therapy (Table 2). Nonetheless, due to the toxicity of chloramphenicol, fluoroquinolones and thiamphenicol, the use of these antibiotics is restricted in neonates. Macrolides might represent a recognized treatment option for *Ureaplasma.* However, macrolide resistance in *Ureaplasma spp.* has been described in different populations [9, 20, 21]. Moreover, with poor CNS penetrance, macrolide antibiotics, such as erythromycin, might not constitute an effective treatment in the case of *U. parvum* meningitis. Regardless, in our case, *Ureaplasma* meningitis was improved by a single drug (erythromycin), as in the case reported by Wang Q et al. [14]. Clifford V also found that combined therapy of erythromycin and ciprofloxacin was effective for neonatal *U. parvum* meningitis [12]. Thus, considering the potential side effects of chloramphenicol, fluoroquinolones and thiamphenicol, as based on limited literature, treatment with erythromycin can be started to determine whether combined therapy needs to be implemented. There is no recommendation about the duration of anti-infection therapy for neonatal *U. parvum* meningitis. We treated our patient with erythromycin for approximately 5 weeks. In other cases, the course of therapy ranged from 3 to 7 weeks.

In conclusion, the manifestation of *U. parvum* meningitis might be similar to that of meningitis due to other bacteria in neonates. When a baby does not respond to regular treatment, detection of *U. parvum* in the CSF should be considered as early as possible. mNGS is recommended, which can identify pathogens in a hypothesis-free way and play an increasingly important role in infectious diseases. The treatment of *U. parvum* meningitis in neonates is still a challenge, and further research is needed.

## Data Availability

The datasets used and/or analysed during the current study are available from the corresponding author on reasonable request.

## Abbreviations

*U. Parvum*: *Ureaplasma parvum*
CSF: Cerebrospinal fluid
mNGS: Metagenomic next-generation sequencing
CNS: Central nervous system
IVH: Intraventricular hemorrhage
VP: Ventriculoperitoneal
PCR: Polymerase chain reaction

## References

[1] Barichello T, Fagundes GD, Generoso JS, Elias SG, Simões LR, Teixeira AL (2013). Pathophysiology of neonatal acute bacterial meningitis. J Med Microbiol.

[2] Gordon SM, Srinivasan L, Harris MC (2017). Neonatal meningitis: overcoming challenges in diagnosis, prognosis, and treatment with Omics. Front Pediatr.

[3] Sprong KE, Mabenge M, Wright CA, Govender S (2020). Ureaplasma species and preterm birth: current perspectives. Crit Rev Microbiol.

[4] Viscardi RM, Kallapur SG (2015). Role of Ureaplasma respiratory tract colonization in Bronchopulmonary dysplasia pathogenesis: current concepts and update. Clin Perinatol.

[5] Waites KB, Katz B, Schelonka RL (2005). Mycoplasmas and ureaplasmas as neonatal pathogens. Clin Microbiol Rev.

[6] Robertson JA, Stemke GW, Davis JW, Harasawa R, Thirkell D, Kong F, Shepard MC, Ford DK (2002). Proposal of Ureaplasma parvum sp. nov. and emended description of Ureaplasma urealyticum (Shepard et al. 1974) Robertson et al. 2001. Int J Syst Evol Microbiol.

[7] Peretz A, Tameri O, Azrad M, Barak S, Perlitz Y, Dahoud WA, Ben-Ami M, Kushnir A (2020). Mycoplasma and Ureaplasma carriage in pregnant women: the prevalence of transmission from mother to newborn. BMC Pregnancy Childbirth.

[8] Kong F, Ma Z, James G, Gordon S, Gilbert GL (2000). Species identification and subtyping of Ureaplasma parvum and Ureaplasma urealyticum using PCR-based assays. J Clin Microbiol.

[9] Glaser K, Speer CP (2015). Neonatal CNS infection and inflammation caused by Ureaplasma species: rare or relevant?. Expert Rev Anti-Infect Ther.

[10] Silwedel C, Haarmann A, Fehrholz M, Claus H, Speer CP, Glaser KJ (2019). More than just inflammation: Ureaplasma species induce apoptosis in human brain microvascular endothelial cells. Neuroinflammation..

[11] Clifford V, Tebruegge M, Everest N, Curtis N (2010). Ureaplasma: pathogen or passenger in neonatal meningitis?. Pediatr Infect Dis J.

[12] Biran V, Dumitrescu AM, Doit C, Gaudin A, Bébéar C, Boutignon H, Bingen E, Baud O, Bonacorsi S, Aujard Y (2010). Ureaplasma parvum meningitis in a full-term newborn. Pediatr Infect Dis J.

[13] Keus AMJMH, Peeters DD, Bekker VV, Veldkamp KEKE, Lambregts MM, Bolt-Wieringa JJ (2019). Neonatal meningitis and subdural empyema caused by an unusual pathogen. Pediatr Infect Dis J.

[14] Wang Q, Wang K, Zhang Y, Lu C, Yan Y, Huang X, Zhou J, Chen L, Wang D (2020). Neonatal Ureaplasma parvum meningitis: a case report and literature review. Transl Pediatr.

[15] Glaser K, Wohlleben M, Speer CP (2015). An 8-month history of meningitis in an extremely low birth weight infant? - long-lasting infection with Ureaplasma parvum. Z Geburtshilfe Neonatol.

[16] Waites KB, Xiao L, Paralanov V, Viscardi RM, Glass JI (2012). Molecular methods for the detection of mycoplasma and ureaplasma infections in humans: a paper from the 2011 William Beaumont Hospital symposium on molecular pathology. J Mol Diagn.

[17] Cao X, Wang Y, Hu X, Qing H, Wang H (2007). Real-time TaqMan polymerase chain reaction assays for quantitative detection and differentiation of Ureaplasma urealyticum and Ureaplasma parvum. Diagn Microbiol Infect Dis.

[18] Ramachandran PS, Wilson MR (2020). Metagenomics for neurological infections - expanding our imagination. Nat Rev Neurol.

[19] Miao Q, Ma Y, Wang Q, Pan J, Zhang Y, Jin W (2018). Microbiological Diagnostic Performance of Metagenomic Next-generation Sequencing When Applied to Clinical Practice. Clin Infect Dis.

[20] Chung HY, Chung JW, Chun SH, Sung HS, Kim MN, Kim KS (2007). A case of erythromycin-resistant Ureaplasma urealyticum meningitis in a premature infant. Korean J Lab Med.

[21] Beeton ML, Spiller OB (2017). Antibiotic resistance among Ureaplasma spp. isolates: cause for concern?. J Antimicrob Chemother.

